# Effect of efavirenz on levonorgestrel concentrations among Malawian levonorgestrel implant users for up to 30 months of concomitant use: a subanalysis of a randomized clinical trial^☆^^☆☆^

**DOI:** 10.1016/j.conx.2020.100027

**Published:** 2020-05-29

**Authors:** Jennifer H. Tang, Nicole L. Davis, Amanda H. Corbett, Lameck Chinula, Mackenzie L. Cottrell, Yasaman Zia, Gerald Tegha, Frank Z. Stanczyk, Stacey Hurst, Mina C. Hosseinipour, Lisa B. Haddad, Athena P. Kourtis

**Affiliations:** a University of North Carolina at Chapel Hill, 111 Mason Farm Road, CB #7577; Chapel Hill, NC, 27599-7577, USA; b UNC Project Malawi, 100 Mzimba Drive, Private Bag A104, Lilongwe, Malawi; c US Centers for Disease Control and Prevention, 1600 Clifton Road, Atlanta, GA, 30329, USA; d University of Southern California, Keck School of Medicine, Reproductive Endocrine Research Laboratory, 1321 N. Mission Road, Los Angeles, CA, 90033, USA; e Emory University School of Medicine, 100 Woodruff Circle, Atlanta, GA, 30322, USA

**Keywords:** Levonorgestrel, Contraceptive implant, Efavirenz, Drug concentrations, Drug interactions

## Abstract

**Objectives:**

Our primary objective was to compare geometric mean levonorgestrel concentrations between levonorgestrel implant users who were or were not taking the antiretroviral efavirenz, for up to 30 months after implant initiation. Our secondary objective was to evaluate the pregnancy rate among levonorgestrel implant users on efavirenz.

**Study design:**

We performed a subanalysis of 42 Malawian women randomized to initiate the levonorgestrel implant as part of a parent randomized clinical trial. Our subset included 30 HIV-infected women taking efavirenz and 12 HIV-uninfected women not taking efavirenz. They underwent urine pregnancy testing every 3 months and serum levonorgestrel testing at day 3 and months 1, 3, 6, 12, 18, 24, 27 and 30 after implant initiation. Geometric mean levonorgestrel concentrations were calculated for efavirenz users and non-efavirenz users at each time point.

**Results:**

The geometric mean levonorgestrel concentrations were lower for efavirenz users than non-efavirenz users at every time point; the geometric mean ratio for efavirenz users:non-efavirenz users ranged from 0.60 [90% confidence interval (CI) 0.46–0.79] at 1 month to 0.27 (90% CI 0.12–0.61) at 30 months after implant insertion. No pregnancies occurred over 60 woman-years of concomitant levonorgestrel implant and efavirenz use, although 11 women had levonorgestrel concentrations < 180 pg/mL (the previously suggested minimum threshold concentration for efficacy).

**Conclusions:**

Efavirenz users had lower levonorgestrel concentrations than non-efavirenz users, and one third of our concomitant efavirenz and levonorgestrel implant users had concentrations < 180 pg/mL. Continued evaluation of the contraceptive efficacy of the levonorgestrel implant may be needed for efavirenz users.

**Implications:**

Among 42 Malawian women using the levonorgestrel implant for contraception, women who were taking the antiretroviral efavirenz had lower serum levonorgestrel concentrations than women who were not taking efavirenz. However, none of the women who were taking efavirenz became pregnant over 60 women-years of follow-up.

## 1 Introduction

HIV-infected women have high rates of unintended pregnancy [[1], [2], [3], [4]]. Contraceptive implants could help them achieve their reproductive goals as implants have pregnancy rates of < 1% during the first year of use [5]. In sub-Saharan Africa (SSA), where most HIV-infected women live, implant use has been rapidly rising. Implants have become the first or second most widely used contraceptive in at least 10 SSA countries [6].

However, studies have found that the antiretroviral efavirenz reduces implant hormone concentrations, which could lead to decreased contraceptive effectiveness. A study of HIV-infected Ugandan women initiating the levonorgestrel implant found that efavirenz users had significantly lower geometric mean levonorgestrel concentrations at weeks 1, 4, 12, 24, 36 and 48 after implant initiation than antiretroviral therapy (ART)-naïve women [7]. In addition, three (15%) pregnancies occurred among 20 efavirenz users between weeks 36 and 48, whereas no pregnancies occurred among 17 ART-naïve women. Two pregnancies occurred among women who had levonorgestrel concentrations above the previously suggested minimum contraceptive threshold of 180 pg/mL at their prior visit [8]. This finding led the authors to evaluate the number of participants who had levonorgestrel concentrations below the highest levonorgestrel concentration at which a pregnancy occurred (303 pg/mL) to assess if a different threshold existed for efavirenz users. However, they found that 18 (90%) of the 20 efavirenz users had levonorgestrel concentrations < 303 pg/mL by week 12 after implant initiation, and 2 (11.8%) of 17 women in the ART-naïve group also had a concentration < 303 pg/mL, which did not support that hypothesis.

Although dolutegravir-based ART is now recommended as the preferred first-line regimen for people living HIV, efavirenz-based ART is an alternative first-line treatment for HIV-infected women in many countries where the implant is used [9]. Therefore, we sought to further characterize drug–drug interactions between the levonorgestrel implant and efavirenz in a subanalysis of a cohort of Malawian women who were randomized to the implant as part of a parent randomized clinical trial. Our primary objective was to compare the geometric mean levonorgestrel concentrations between levonorgestrel implant users who were or were not taking efavirenz, for up to 30 months after implant initiation. Our secondary objective was to evaluate the pregnancy rate among levonorgestrel implant users on efavirenz.

## 2 Material and methods

### 2.1 Study recruitment, enrollment and visits

The main randomized clinical trial and this subanalysis were approved by the University of North Carolina (UNC) and U.S. Centers for Disease Control and Prevention Institutional Review Boards (IRBs), the Malawi National Health Sciences Research Committee, and the Malawi Pharmacy Medicines and Poisons Board. The study procedures for the main trial have been previously described [10]. Participants were recruited from the Family Planning Clinic at Bwaila Hospital in Lilongwe, Malawi, the site of all study visits. Women interested in study participation provided written informed consent for study screening. Inclusion criteria were (1) age 18–45 years; (2) known HIV status; (3) at least 2 regular, monthly cycles in the 3 months preceding study enrollment; (4) no hormonal or intrauterine contraceptive use for at least 6 months prior to enrollment; (5) at least 6 months since pregnancy; (6) interested in initiating the depot medroxyprogesterone acetate (DMPA) injectable or the levonorgestrel implant; (7) willing to be randomized to either DMPA or the levonorgestrel implant and (8) willing to wait 4–6 weeks after enrollment to receive the method and to use nonhormonal or nonintrauterine methods during this period. Exclusion criteria were (1) pregnancy, (2) desire to become pregnant within the next 12 months, (3) World Health Organization Medical Eligibility Criteria for Contraceptive Use Category 3 or 4 condition for DMPA or levonorgestrel implant [11], (4) known acute HIV infection or (5) history of tubal ligation or hysterectomy.

Eligible women were consented for enrollment and then completed a baseline interview. They were scheduled to complete visit 1 during the first 14 days of their next anticipated menstrual cycle and then visit 2 between day 15 from the onset of last menses until start of next menses. Women were then randomized to initiate DMPA or the levonorgestrel implant at visit 3, which occurred during the first 7 days after the start of the next menses. Women were scheduled to return on day 3 (visit 4) and then months 1, 3, 6, 9, 12, 15, 18, 21, 24, 27, 30 and 33 (visits 5–16) after randomization. Since women were randomized to their contraceptive between May 2014 and April 2015 and the study ended in May 2017, most women did not complete all 33 months of follow-up after randomization and were exited at an earlier study visit. In addition, the study was initially planned to follow women for only 6 months after randomization, and a gap in following up some women occurred during the month 9, 12, and 15 postrandomization visits while awaiting IRB approval to extend the study.

If a woman was randomized to the levonorgestrel implant, she had the two-rod implant (levonorgestrel 75 mg/rod; Jadelle®, Bayer AG, Berlin, Germany) inserted and then had it palpated at every follow-up visit to confirm its continued use. At the end of every study visit, we read a counseling script which advised women to use condoms if they thought they were at risk of getting HIV or giving HIV to their partner and offered them condoms.

In December 2014, we submitted an amendment to all four regulatory boards to update our study protocol, consent forms, implant counseling aid and end-of-visit counseling script to include recently published information about the potential decreased effectiveness of the levonorgestrel implant among HIV-infected women on ART and the need to use condoms for back-up contraception [12,13]. Three separate implant counseling aids were developed to ensure that women could make informed decisions about (1) whether to enroll (if undergoing study screening), (2) whether to continue in the study (if enrolled but not yet randomized) and (3) whether to continue with the implant (if already randomized to the implant). Amendment approval from all IRBs was received by the end of March 2015, and the revised documents were implemented immediately thereafter.

At each visit, we completed an Interval History Form and Physical Exam Form to confirm her medications and to assess for sexual activity and condom use since her last study visit. To assess for sexual activity and condom use, we asked “Have you had vaginal sex since your last visit with us? (Yes/No/Do Not Know).” If she answered yes, she was asked “Since your last visit, how often did your partner(s) use a condom when you had vaginal sex? (Always/Sometimes/Never/Decline to Respond/Do Not Know).” To assess for pregnancy, we performed urine pregnancy testing at the screening/enrollment visit, visit 3 and then at 3-monthly visits until 33 months after contraceptive initiation. At each visit, we also asked the question “Since your last visit, have you had a positive pregnancy test or been told that you were pregnant?” However, we only collected blood samples from the participants at the following visits: day 3 and months 1, 3, 6, 12, 18, 24, 27, 30 and 33 after contraceptive initiation.

### 2.2 Blood sample collection and ART and hormone analysis

For the blood collection, both a 6-mL red-top tube and a 5-mL purple-top tube were collected and transported on ice to the UNC Project-Malawi Laboratory, where they were immediately centrifuged. Serum from the red-top tubes and plasma from the purple-top tubes were aliquoted into 1-mL cryovials, purposed for levonorgestrel and efavirenz quantification, respectively, and then stored at − 80°C until the time of analysis.

### 2.3 Bioanalytical methods

Efavirenz was quantified in plasma by the UNC Center for AIDS Research Clinical Pharmacology and Analytical Chemistry Core using validated high-performance liquid chromatography (HPLC)–tandem mass spectrometry (MS) methods. Following protein precipitation of 30 μL of plasma, efavirenz was extracted with an isotopically labeled internal standard (efavirenz-d_5_) using reverse-phase chromatography on a Waters Atlantis T3 (50 × 2.1 mm, 3 μm) analytical column. Standards and quality controls were prepared in blank human plasma or blank 70:30 methanol:water to achieve a calibrated range of 50–20,000 ng/mL, with ± 15% (20% at the lower limit of quantification) precision and accuracy acceptance criteria.

Levonorgestrel was quantified in serum by the University of Southern California Reproductive Endocrine Research Laboratory in Los Angeles, CA, by a specific and sensitive radioimmunoassay (RIA) [14]. Prior to RIA, levonorgestrel was extracted with ethyl acetate:hexane (3:2) and subjected to Celite column partition chromatography. Procedural losses were followed by adding approximately 800 dpm of high specific activity tritiated internal standard (^3^H-levonorgestrel) to the serum prior to the extraction step. A highly specific antiserum was used in conjunction with an iodinated radioligand in the RIA. Separation of free from antiserum-bound levonorgestrel was achieved by use of a second antibody. The lower limit of levonorgestrel detection was 0.025 ng/mL, with intra-assay and interassay coefficients of variation of 4.4% and 8.9% [14].

### 2.4 Pharmacokinetic and statistical analyses

Participant baseline characteristics were compared between efavirenz users and non-efavirenz users using the Wilcoxon test for continuous data and the *χ^2^* test for categorical data. Women were censored from their group if the implant was discontinued or if they were switched to an ART regimen that did not include efavirenz (if they were previously an efavirenz user); no women switched from the nonefavirenz group to the efavirenz group. We calculated the geometric mean levonorgestrel concentrations with 90% confidence intervals (CIs) for each time point after implant initiation except for month 33 (due to small numbers). We then calculated the geometric mean ratio (GMR) and 90% CI for efavirenz users:non-efavirenz users at each time point. In addition, we calculated the difference in the individual levonorgestrel values at each time point using Wilcoxon Mann–Whitney exact tests due to the small number of non-efavirenz users.

We also calculated levonorgestrel area under the curve for months 0–6 after implant initiation (AUC_0__–__6 months_) for both efavirenz users and non-efavirenz users and used the Wilcoxon Mann–Whitney exact test to compare their curves. We only calculated AUC_0__–__6 months_ because the 6-monthly samples that we collected after month 6 were too far apart to allow us to apply the trapezoidal rule for AUC calculation. To evaluate for correlations between serum levonorgestrel concentrations and plasma efavirenz concentrations, we used Spearman's correlation coefficient. Woman-years of follow-up were determined by calculating the number of days between implant insertion and the last date that the woman was confirmed to be taking efavirenz and using the implant for each of the 30 women in the efavirenz group. The total number of days of concomitant exposure was then summed up for the 30 women and divided by 365 days to convert the woman-days to woman-years.

## 3 Results

Between May 2014 and April 2015, we randomized 73 HIV-positive women (37 to DMPA and 36 to the levonorgestrel implant) and 24 HIV-negative women (12 to DMPA and 12 to the implant). For this subanalysis, we analyzed 42 women (30 HIV-infected, 12 uninfected) randomized to initiate the implant. All 30 HIV-infected women were using combination ART consisting of daily efavirenz 600 mg, tenofovir disoproxil fumarate 300 mg and lamivudine 300 mg, whereas none of the 12 uninfected women were taking efavirenz.

The median age was 34 years for efavirenz users and 26 years for non-efavirenz users, and the median weight was 57 kg for efavirenz users and 54 kg for non-efavirenz users (Table 1). Fifteen (52%) efavirenz users reported having sex since their last visit at 76%–100% of visits; 12 (41%) reported that they always used condoms since the last visit at 76–%100% of visits. No study participants took rifampicin or any other medications that interact with efavirenz or levonorgestrel.

**Table 1:** Participant characteristics of the 48 Malawian women who initiated the levonorgestrel implant, stratified by efavirenz use

Participant characteristics^a^	Efavirenz nonusers (*n* = 12)	Efavirenz users (*n* = 30)
Median age (years)	26 (23–31)	34 (29–38)
Weight (kg)	54 (49–59)	57 (53–64)
HIV-infected, *n* (%)	0 (0)	30 (100)
Median number of living children	2 (2–3)	2.5 (2–3)
Married, *n* (%)	10 (83)	19 (63)
Less than primary education, *n* (%)	5 (42)	13 (43)
Reported prior use of hormonal contraception, *n* (%)	9 (75)	22 (73)
Reported that they had vaginal sex since last visit, *n* (%)^^b^		
Reported at 0%–25% of visits	1 (8)	4 (14)
Reported at 26%–50% visits	1 (8)	2 (7)
Reported at 50%–75% visits	2 (17)	8 (28)
Reported at 76%–100% visits	8 (67)	15 (52)
Among those that reported vaginal sex since last visit, reported that they always used a condom since last visit^^b^		
Reported at 0%–25% of visits	9 (75)	4 (14)
Reported at 26%–50% visits	2 (17)	8 (28)
Reported at 50%–75% visits	0 (0)	5 (12)
Reported at 76%–100% visits	1 (8)	12 (41)
**For HIV-infected women only:**	***n*** **=** **0**	***n*** **=** **30**
Time since HIV diagnosis (years)	N/A	
< 5 years		19 (63)
5–10 years		9 (30)
> 10 years		2 (7)
Time on antiretroviral medication (years)^c^	N/A	
< 1 year		5 (17)
1–5 years		17 (59)
> 5 years		7 (24)
Median CD4 + T-cell count	N/A	282 (194–451)
Median HIV viral load	N/A	0 (0–1087)

Data are presented as either median (interquartile range) or *n* (%), as appropriate.

a Data were obtained from baseline interview unless marked with ^.

b Response missing for one efavirenz user because she never responded to these questions.

c Time on antiretroviral medication was missing for one efavirenz user.

Three efavirenz users discontinued their implant between months 3 and 6 and so were censored after month 3. Another efavirenz user discontinued her implant between months 6 and 9 and so was censored after month 6, and a final efavirenz user discontinued her implant between months 18 and 21 and so was censored after month 18. Three of the HIV-uninfected women discontinued their implants by months 18, 21 and 24 and so were censored after the visit immediately preceding implant discontinuation. Finally, another two efavirenz users were switched to second-line ART (one before month 3, the other before month 6), so they were censored at the visit immediately preceding ART switch.

**Fig. 1:**
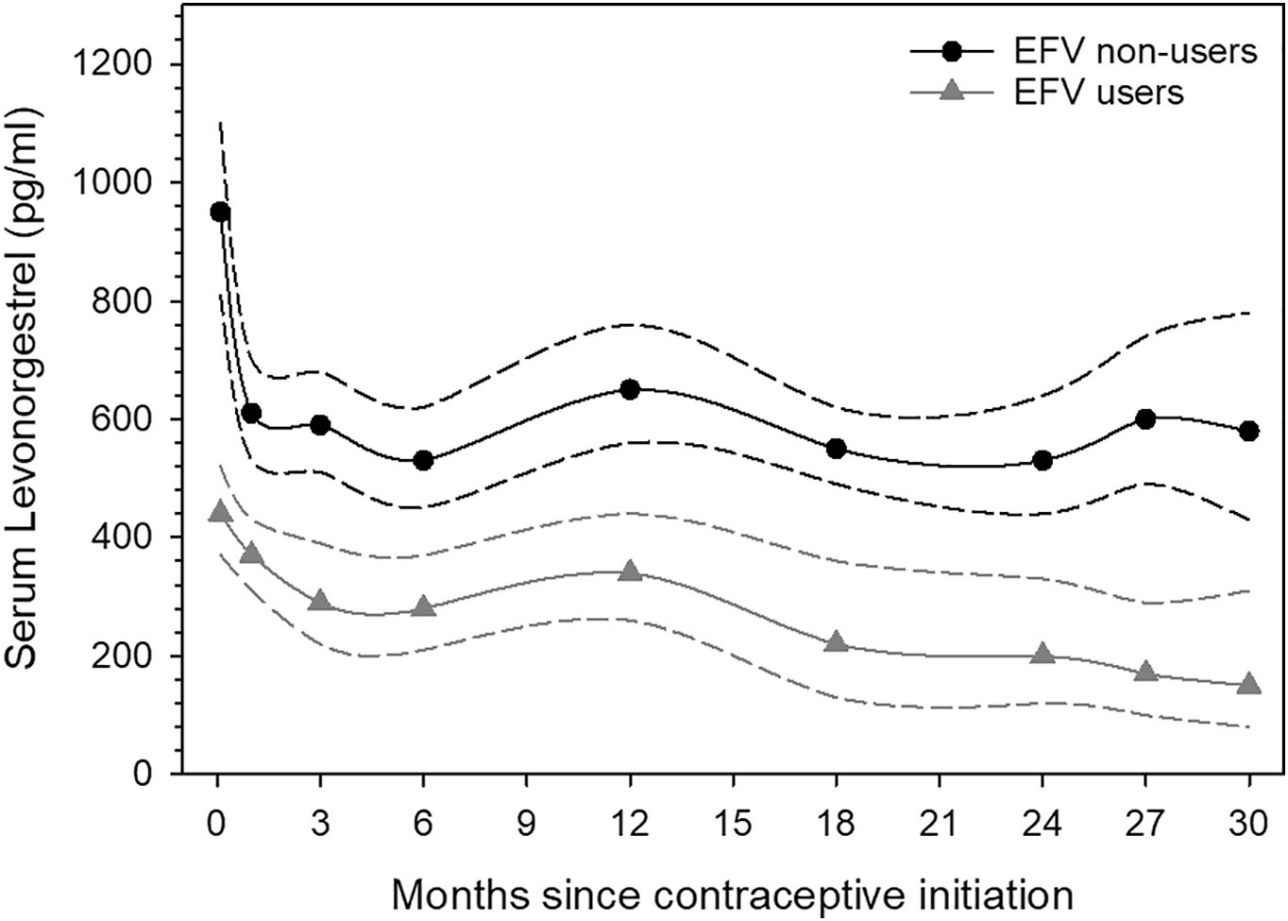
Graphical comparison of geometric mean serum levonorgestrel concentrations by time since levonorgestrel implant initiation between efavirenz users and efavirenz nonusers. Legend: EFV = efavirenz.

The geometric mean levonorgestrel concentrations were significantly lower in efavirenz users than the geometric mean levonorgestrel concentrations for non-efavirenz users at every time point (Fig. 1, Table 2). The GMR for efavirenz users:non-efavirenz users ranged from 0.60 (90% CI 0.46–0.79) at 1 month to 0.27 (90% CI 0.12–0.61) at 30 months after implant insertion. Likewise, the geometric mean (90% CI) levonorgestrel area under the curve (AUC_0__–__6 months_) was significantly lower in efavirenz users [8.44 (90% CI 7.03–10.13 h*ng/mL) than the geometric mean levonorgestrel area under the curve (AUC_0__–__6 months_) for non-efavirenz users [14.54 (90% CI 12.67–16.70 h*ng/mL). The AUC geometric mean ratio (90% CI) for efavirenz users:non-efavirenz users was 0.58 (90% CI 0.46–0.73) (p = .002).

**Table 2:** A comparison of the geometric mean levonorgestrel serum concentrations by time since levonorgestrel implant initiation between efavirenz users and efavirenz nonusers

	Efavirenz nonusers		Efavirenz users		Efavirenz users/efavirenz nonusers	
Months from contraceptive initiation	*n*	Geometric mean levonorgestrel^a^ (90% CI)	*n*	Geometric mean levonorgestrel^a^ (90% CI)	Geometric mean ratio (90% CI)	p value^b^
Day 3	12	950 (810–1110)	29	440 (370–520)	0.47 (0.35–0.61)	< .0001
1	12	610 (530–700)	30	370 (310–430)	0.60 (0.46–0.79)	.002
3	12	590 (510–680)	27	290 (220–390)	0.50 (0.33–0.76)	.001
6	12	530 (450–620)	26	280 (210–370)	0.53 (0.34–0.82)	.004
12	5	650 (560–760)	11	340 (260–440)	0.52 (0.34–0.78)	.008
18	10	550 (490–620)	22	220 (130–360)	0.40 (0.19–0.84)	.01
24	8	530 (440–640)	18	200 (120–330)	0.39 (0.18–0.81)	.007
27	7	600 (490–740)	12	170 (100–290)	0.28 (0.14–0.58)	.0004
30	5	580 (430–780)	7	150 (80–310)	0.27 (0.12–0.61)	.01

a In pg/mL.

b Based on Wilcoxon Mann–Whitney exact test due to small number of efavirenz nonusers.

By month 27, the geometric mean levonorgestrel concentration for efavirenz users had fallen below the previously suggested contraceptive threshold of 180 pg/mL [8]. However, no pregnancies occurred over 60 woman-years of concomitant implant and efavirenz use, even though 11 (36.7%) of the 30 women had levonorgestrel concentrations of < 180 pg/mL during at least one time point, ranging from as early as 3 days to 547 days after implant initiation.

**Fig. 2:**
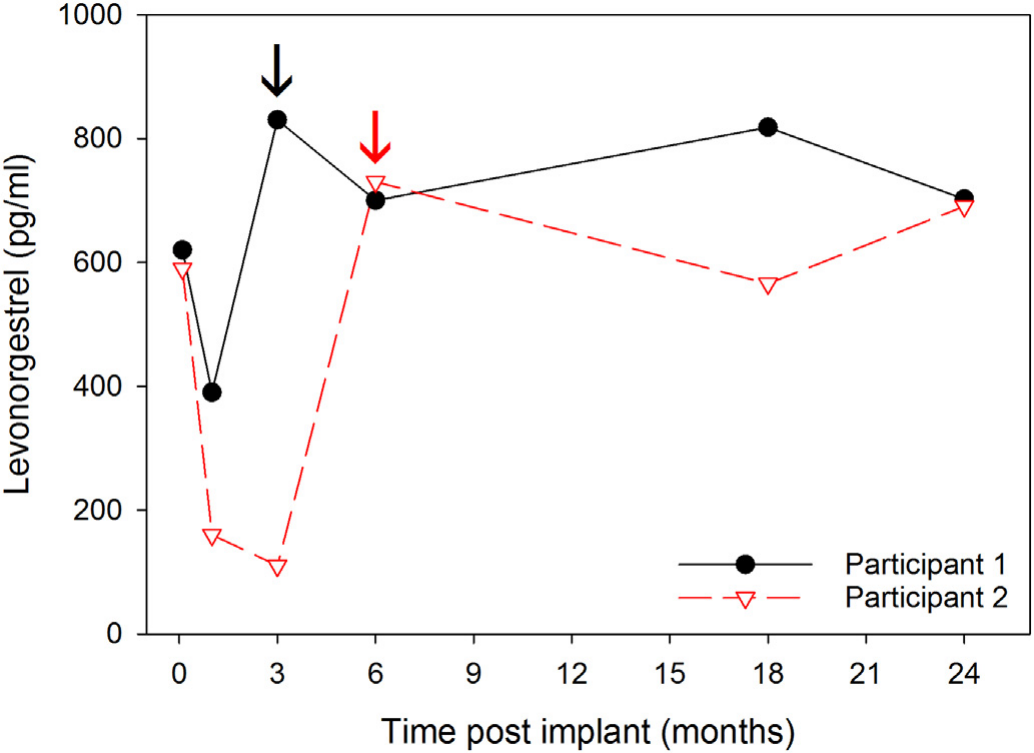
Levonorgestrel concentrations rebound after stopping efavirenz among two HIV-infected levonorgestrel implant users who switched to a nonefavirenz-based antiretroviral therapy regimen. Legend: Levonorgestrel concentrations for two users undergoing switching to a nonefavirenz-based antiretroviral therapy regimen after having received the implant are plotted over time. Color-coded arrows correspond to the first study visit occurring after the switch from efavirenz. In both instances, serum levonorgestrel concentrations rebound above baseline concentrations.

Serum levonorgestrel concentrations were inversely correlated with plasma efavirenz concentrations (*r*^2^ = − 0.595, p < .001). Given the relationship between levonorgestrel and efavirenz concentrations, we examined the levonorgestrel concentrations of the two efavirenz users who switched to a non-efavirenz regimen before and after the ART change (Fig. 2). For the first participant (participant 1), levonorgestrel concentration dropped from 620 pg/mL to 390 pg/mL between day 3 and month 1 after contraceptive initiation while on efavirenz. However, she switched to a nonefavirenz regimen prior to month 3, at which time her levonorgestrel concentration increased to 830 pg/mL, and it remained above 700 pg/mL until her last visit at month 24. The second participant (participant 2) had a similar trajectory: her levonorgestrel concentration dropped from 590 pg/mL to 110 pg/mL between day 3 and month 3 after contraceptive initiation while on efavirenz. She then switched to a nonefavirenz regimen prior to month 6, at which time her levonorgestrel level increased to 730 pg/mL. It remained above 567 pg/mL until her last visit at month 24. These postefavirenz levonorgestrel concentrations were similar to the geometric mean month 24 levonorgestrel concentrations of the non-efavirenz users, which was 590 pg/mL (90% CI 500–690), and indicate that, in women who switch from efavirenz to other antiretroviral drugs, levonorgestrel concentrations rebound to levels expected for non-efavirenz users.

## 4 Discussion

Our results show a reduction in levonorgestrel concentrations among efavirenz users as compared to non-efavirenz users starting immediately after insertion. Although one third of our concomitant efavirenz and levonorgestrel implant users had concentrations < 180 pg/mL, no pregnancies occurred in this study over 60 women-years of follow-up. This latter finding contrasts with the Ugandan study, which had a 15% pregnancy rate within 48 weeks of implant insertion among 20 efavirenz users [7], and with a retrospective study from Swaziland that found that 15 of 121 (12.4%) efavirenz users who initiated the levonorgestrel implant became pregnant at a mean duration of 16.4 months [12]. Our pregnancy rate is also lower than a larger retrospective cohort study of almost 25,000 HIV-infected Kenyan women, which found a pregnancy rate of 7.1 per 100 woman-years (4.3 per 60 woman-years) for efavirenz users who initiated the levonorgestrel implant [15,16].

Our study is similar to the Ugandan study in that it found that the levonorgestrel GMR for efavirenz users:ART-naïve women ranged from 0.43 to 0.55 between weeks 1 and 48 after implant initiation. In addition, we found similar levonorgestrel concentrations among efavirenz users at weeks 4, 12, 24 and 48, even though we used RIA and they used HPLC–MS [7]. The age, weight and time on ART are also comparable between the two studies, but the women in our study had a median CD4 + count (282 mm^3/^mL) about half that of the women in the Ugandan study (557 mm^3/^mL). The difference in pregnancy rates between the studies could be due to poorer health among our women or to differences in sexual frequency and condom use, neither of which was reported in the Ugandan study. Our efavirenz users reported high rates of condom use, possibly because we provided condoms at each visit and performed extensive counseling at each visit about the need to use condoms. However, condom use was not consistent, and another subanalysis from this study found that discordance between prostate-specific antigen test results (a marker of recent sexual activity) and self-report of sexual activity was more prevalent among HIV-infected women in our cohort [17].

Our finding that serum levonorgestrel concentrations were inversely correlated with plasma efavirenz concentrations suggests that women with slower metabolism of efavirenz have lower concentrations of levonorgestrel. Previous pharmacogenetic studies performed in Malawi and other countries have found that efavirenz concentrations are higher in African efavirenz users compared to non-African efavirenz users [18,19] and that genetic variants are associated with decreased levonorgestrel concentrations among efavirenz users [20,21]. Further research to evaluate the role of genetic variants on efavirenz and levonorgestrel concentrations and their effects on contraceptive efficacy might allow development of targeted recommendations for different individuals.

In addition, we demonstrated that levonorgestrel concentrations among efavirenz users rebounded to similar concentrations as non-efavirenz users after efavirenz was discontinued, so women nonadherent to efavirenz would presumably have higher levonorgestrel concentrations during periods of nonadherence. Future studies could use hair samples to better evaluate the longitudinal effect of efavirenz exposure on levonorgestrel concentrations and unintended pregnancy among implant users; hair concentrations are a more accurate biomarker of long-term efavirenz adherence and metabolism than blood concentrations, which can only capture efavirenz concentrations at a single point in time [22].

Strengths of our study include the length of follow-up for up to 33 months after implant initiation and that we were able to evaluate the effect of efavirenz discontinuation on levonorgestrel concentrations among two women who discontinued use of the implant. In addition, we evaluated and showed a strong negative correlation between levonorgestrel and efavirenz concentrations. However, our sample size for time points beyond 6 months after implant initiation was smaller, although we were able to evaluate levonorgestrel concentrations on > 60% of participants in both the efavirenz and nonefavirenz groups at months 18 and 24. The lack of observed pregnancies may have been due, in part, to our small sample size, although the Ugandan study only had 20 efavirenz users [7]. In addition, we could have missed pregnancies between study visits if a participant aborted and did not report the pregnancy at her next visit.

In summary, our subanalysis adds important new knowledge about the long-term effect of efavirenz on levonorgestrel concentrations, including how levonorgestrel concentrations respond after efavirenz discontinuation. Although some women may now be switching from efavirenz-based ART to dolutegravir-based ART based on the most recent WHO 2019 recommendations, since efavirenz-based ART is still considered an alternative first-line ART, efavirenz will continue to be used for now by reproductive-age HIV-infected women [9]. We hope that our results will help policymakers to weigh the risks and benefits of concomitant use of implants while on efavirenz-based ART and stimulate further research to understand thresholds for contraceptive efficacy and implant failure.
